# Improved *p*-type conductivity in Al-rich AlGaN using multidimensional Mg-doped superlattices

**DOI:** 10.1038/srep21897

**Published:** 2016-02-24

**Authors:** T. C. Zheng, W. Lin, R. Liu, D. J. Cai, J. C. Li, S. P. Li, J. Y. Kang

**Affiliations:** 1 Department of Physics, OSED, Fujian Provincial Key Laboratory of Semiconductor Materials and Applications, Xiamen University, Xiamen, 361005, China; 2 SIMS Lab, Office of the Deputy Vice Chancellor and Vice President, Research and Development, Western Sydney University, Locked Bag 1797 Penrith NSW 2751

## Abstract

A novel multidimensional Mg-doped superlattice (SL) is proposed to enhance vertical hole conductivity in conventional Mg-doped AlGaN SL which generally suffers from large potential barrier for holes. Electronic structure calculations within the first-principle theoretical framework indicate that the densities of states (DOS) of the valence band nearby the Fermi level are more delocalized along the *c*-axis than that in conventional SL, and the potential barrier significantly decreases. Hole concentration is greatly enhanced in the barrier of multidimensional SL. Detailed comparisons of partial charges and decomposed DOS reveal that the improvement of vertical conductance may be ascribed to the stronger *p*_*z*_ hybridization between Mg and N. Based on the theoretical analysis, highly conductive *p*-type multidimensional Al_0.63_Ga_0.37_N/Al_0.51_Ga_0.49_N SLs are grown with identified steps via metalorganic vapor-phase epitaxy. The hole concentration reaches up to 3.5 × 10^18^ cm^−3^, while the corresponding resistivity reduces to 0.7 Ω cm at room temperature, which is tens times improvement in conductivity compared with that of conventional SLs. High hole concentration can be maintained even at 100 K. High *p*-type conductivity in Al-rich structural material is an important step for the future design of superior AlGaN-based deep ultraviolet devices.

Al-rich (Al > 0.5) AlGaN alloys are a promising class of materials because they are especially suited for realizing deep ultraviolet optoelectronic devices with operating wavelength down to 200 nm^1^,^2^,^3^,^4^. Although *n*-type layers can be produced relative easily^5^,^6^,^7^,^8^,^9^, achieving desirable conductivity for *p*-type Al_*x*_Ga_1−*x*_N with *x* > 0.5 is still a significant challenge^10^,^11^,^12^,^13^. Magnesium is commonly used as *p*-type dopant, but its doping efficiency is still far from satisfactory due to a combination of factors involving limited solubility, high activation energy, increased donor compensations, and increased hole scatterings^14^,^15^. The Mg limited solubility, which is caused by the high formation enthalpies of Mg substitution for Ga or Al, becomes increasingly obvious as Al content increases^16^. To enhance the effective incorporation of Mg, we previously proposed a modified surface-engineering technique that applies periodical interruptions under an ultimate V/III ratio condition (extremely N-rich)^16^. Apart from the solubility limit, the activation energy of an Mg acceptor in Al_*x*_Ga_1−*x*_N alloys increases almost linearly with Al content, from 170 meV to 510 meV with *x* from 0 to 1 10,11. This behavior results in a decreasing acceptor activation efficiency, in which only a very small fraction (~10^−9^) of Mg dopant can be activated at room temperature in Mg-doped AlN^10^. To date, the desirable *p*-type conduction of Al_*x*_Ga_1−*x*_N alloys with Al content over 0.5 is rarely reported^12^,^13^. The resistivity value obtained for *p*-type Al_0.7_Ga_0.3_N is as high as 10^5^ Ω cm at room temperature^12^, hampering device performance with poor hole injection. Thus, unraveling the Mg doping mechanism is crucial for effectively improving the *p*-type conductivity in Al-rich AlGaN.

Enormous efforts have been made to lower Mg activation energy so as to increase doping efficiency. The current state of available *p*-type growth techniques, including Mg–O^17^,^18^, Mg–Zn^19^, or Mg–Si co-doping^20^, delta doping^21^,^22^,^23^, polarization-induced hole doping^24^, and superlattice (SL) doping^25^,^26^,^27^,^28^,^29^, have been speculated to increase the hole concentration in GaN or low Al-content AlGaN films. However, stable *p*-type doping has yet to be experimentally established in Al-rich AlGaN. Most activities at the moment are concentrated on SL doping, where the valence band discontinuity together with innate strong polarization fields along the *c*-axis of the III-N crystal create a periodic oscillation of the valence band edge. Such oscillation leads to a periodic ionization of the acceptor, with holes accumulated where the valence band edge is close to the Fermi energy^25^,^27^. Thus, confined parallel sheets of two-dimensional (2D) hole gases are formed in Mg-doped low Al-content AlGaN/GaN SLs^26^,^28^. Such parallel 2D hole sheets suffer from low conductivity along the *c* vertical axis.because of large potential barriers that require the hole to transport through tunneling or thermionic emission processes caused by strong polarization fields and valence band discontinuity^30^,^31^,^32^,^33^, although they have high in-plane conductivity. Furthermore, hole scatterings become increasingly significant as Al content increases, which aggravates low vertical hole mobility^34^,^35^,^36^. In this case, developing an alternative strategy for efficient *p*-type doping and hole injection in Al-rich AlGaN devices is highly desirable.

In this work, we propose multidimensional SL doping extending in-plane layers of conventional SL doping into higher dimension, which is expected to enhance the vertical hole conductivity. The structure of three-dimensional (3D) AlN/GaN SL as a representative multidimensional model accompanied with conventional AlN/GaN SL is illustrated in Fig. 1. First-principle simulations were utilized to investigate the Mg doping behavior in multidimensional and conventional AlGaN SL. Simulations details are available in the methods section. Based on the theoretical results, multidimensional Al_0.63_Ga_0.37_N/Al_0.51_Ga_0.49_N SLs were applied to enhance the Mg doping efficiency via low-pressure metalorganic vapor-phase epitaxy (MOVPE). *I*-*V* and Hall measurements were conducted to analyze the electrical characteristic. A comparison of the proposed multidimensional SLs with the conventional SLs demonstrated a significant enhancement in Mg doping efficiency and excellent *p*-type conductivity.

## Results

**Figure 2:**
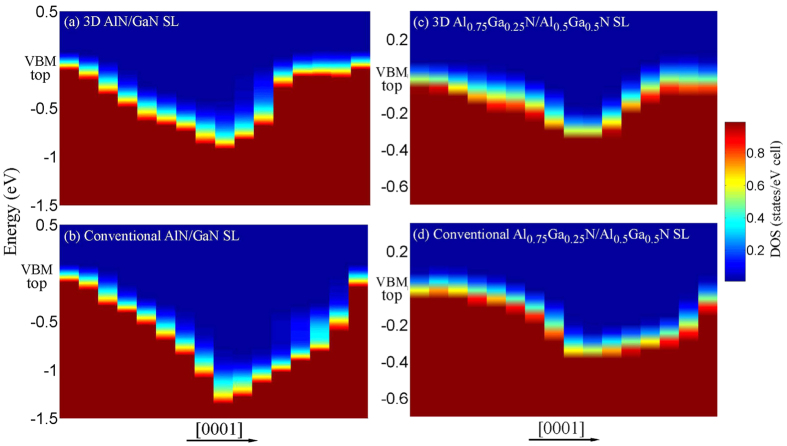
Projected DOS of valence bands along the [0001] direction for each bilayer in undoped 3D AlN/GaN SL (**a**), conventional AlN/GaN SL (**b**) 3D Al_0.75_Ga_0.25_N/Al_0.5_Ga_0.5_N SL (**c**), and conventional Al_0.75_Ga_0.25_N/Al_0.5_Ga_0.5_N SL (**d**).

Valence band discontinuity together with built-in electric fields caused by spontaneous and piezoelectric polarization effects within conventional SL are known to create periodic oscillation of the valence band edge^25^,^27^. Meanwhile, strong built-in electric fields also lead to a large potential barrier hampering the hole to transport through SLs along the *c*-axis^30^,^31^,^32^,^33^. Based on the projected densities of states (DOS) of valence bands along the [0001] direction for each bilayer in Fig. 2, the valence band maximum (VBM) in 3D SL with the largest DOS is located at the interface between well and barrier, similar to conventional SL. However, the valence band edge of barrier significantly uplift, which lowers the potential barrier for vertical hole transport.

**Figure 3:**
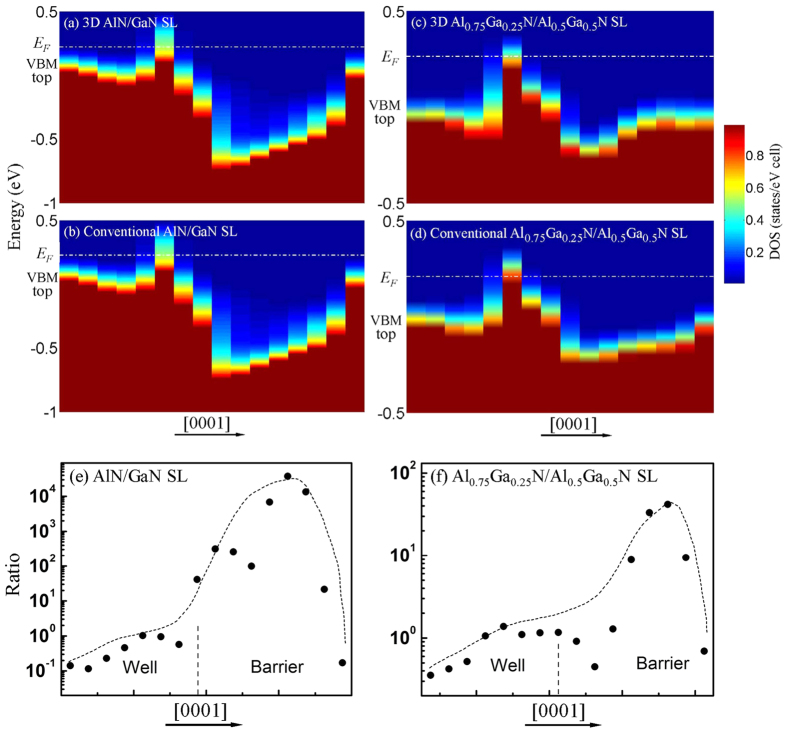
Projected DOS of valence bands along the [0001] direction for each bilayer in Mg-doped 3D and conventional SL (**a–d**). Hole concentration ratio of 3D SL to conventional SL for AlN/GaN (**e**) and Al_0.75_Ga_0.25_N/Al_0.5_Ga_0.5_N (**f**).

By doping Mg in the effective region of 3D SL^25^,^27^, the DOS of the valence band nearby Fermi level (*E*_F_) are more deconcentrated along the *c*-axis than that of conventional SL, and the potential barrier is significantly decreased (Fig. 3a–d). To understand the vertical transport improvement of the hole in 3D SL, we compared the free hole concentrations between 3D and conventional SL in terms of the Fermi distribution^25^,^33^ in conjunction with the DOS of the valence band. Their ratio *R* is given by


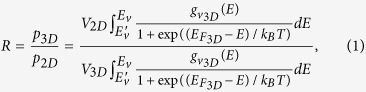


where *g*_*v*_(*E*) is the DOS per supercells, *E* is the energy of the valence band, *V* is the volume of supercells, *k*_*B*_ is the Boltzmann constant, and *T* is the temperature. The calculated ratio is much higher than 1 by several orders in the barrier region, but slightly lower than 1 in the well [Fig. 3e,f]. Although total hole concentrations are nearly similar in the two structures, the hole concentration is enhanced by several thousand times in the barrier of 3D SL. The bottleneck problem of poor vertical conductance in conventional SL is expected to be solved by selectively doping Mg into multidimensional SL.

**Figure 4:**
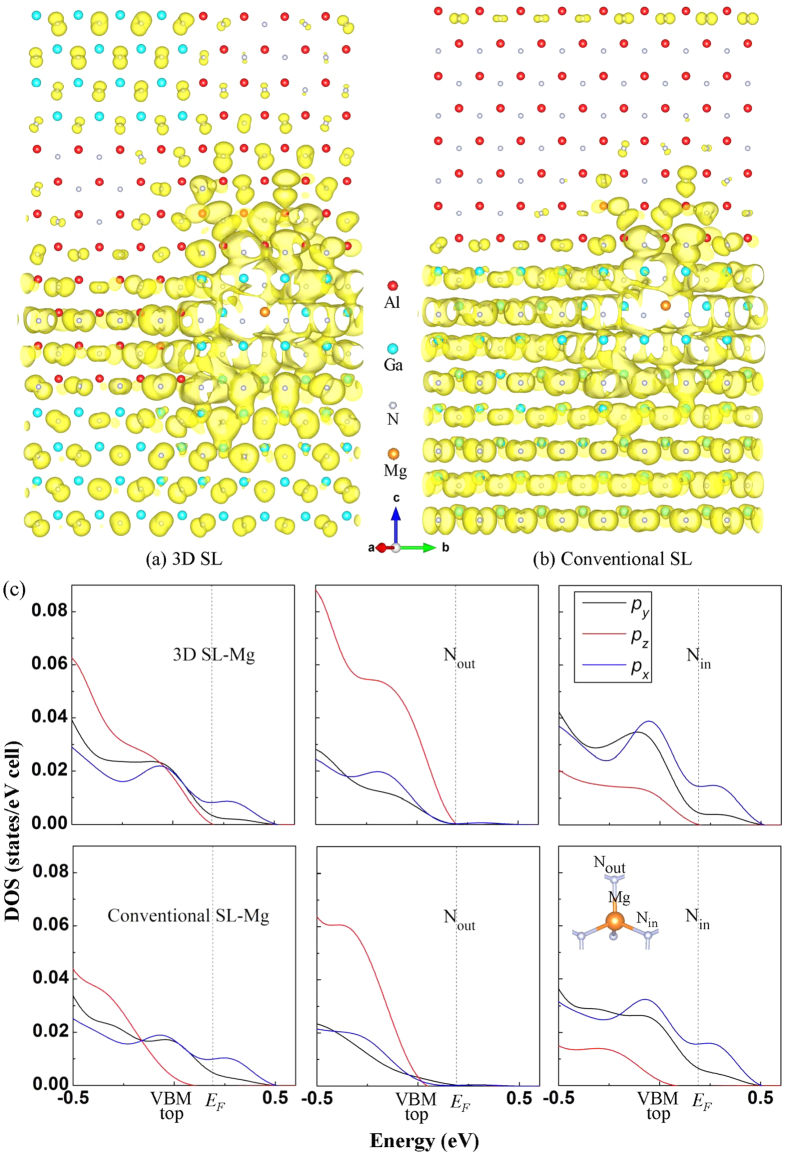
The isosurface of the valence states at the top of valance bands for Mg-doped 3D (**a**) and conventional (**b**) GaN/AlN SLs with k vector restricted to [0001]. (**c**) Decomposed DOS of *p*_*x*_, *p*_*y*_, and *p*_*z*_ of Mg and bonded N atoms in 3D SL and conventional SL. The N bonded with Mg lying in the *ab* plane and out of the plane is respectively denoted as N_in_ and N_out_ in the inset.

Insight into the different behaviors of Mg dopant between two structures is gained by comparing the charge distribution (orbitals) at the top of valance bands with **k** vector restricted to the [0001] direction. The isosurface of the relevant charge densities at the top of valance bands for 3D and conventional GaN/AlN SL are plotted in Fig. 4a,b, respectively. The Mg dopant in 3D SL is closely surrounded by charges of N atoms extending through the entire SL along the *c*-axis while being separated in conventional SL. Therefore, the hole in 3D SL are likely to be more delocalized rather than concentrated in the well compared with that in the conventional SL. The charge distribution is present in the form of an asymmetric orbital, which can be identified as *p* states for a large part. In general, the *p* orbital of N is composed of *p*_*x*_, *p*_*y*_ and *p*_*z*_ states. To clarify the factor that brings in the improved vertical conductance, the site-decomposed DOS of *p*_*x*_, *p*_*y*_ and *p*_*z*_ for Mg and N atoms bonded with Mg are further displayed in Fig. 4c. The site-decomposed DOS of Mg and the bonded N nearby *E*_F_ in 3D SL are much larger than those in conventional SL. Notably, the *p*_*z*_ DOS of Mg and N nearby *E*_F_ is simultaneously enhanced in the 3D SL, suggesting the stronger *p*_*z*_ hybridization between Mg and N. With regard to the charge in Fig. 4a, large part of the N charges primarily oriented to the *c*-axis is likely to be associated with the *p*_z_ states of N. In 3D SL, the lobes of *p*_z_ orbital slightly deviate from the *c*-axis and point toward the Mg dopant, demonstrating radiative interaction with N atoms. Specifically, the higher value in *p*_*z*_ DOS nearby *E*_F_ of N_out_ atoms bonded with Mg is much significant, thus allowing higher vertical hole production.

**Figure 5:**
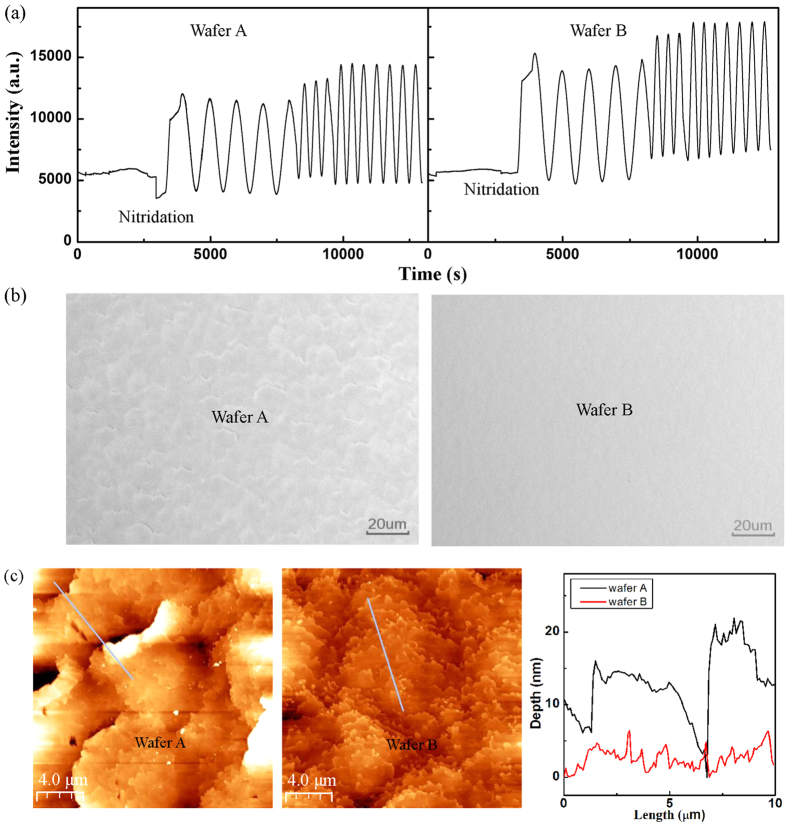
(**a**) *In situ* reflectance curves during growth. Optical microscopic (**b**) and AFM (**c**) image of the AlGaN SLs surface.

In practical growth, multidimensional SLs can be shaped in the form of 3D SLs in different sizes which are likely in favor of improved *p*-type conductivity on account of the simulation results. To demonstrate the effectiveness of multidimensional SLs, two types of structure wafers with Mg-doped AlGaN SLs were grown by MOVPE. Interference spectrum yields *in situ* data enable a close control over growth quality. In the initial growth stage of wafer A, the intensity of the *in situ* interference spectrum significantly decreased, indicating the longer sapphire nitridation time results in the increase of surface roughness [Fig. 5a]. During the subsequent epilayer growth, the oscillating amplitude of wafer A were smaller than that of wafer B. Considering that the oscillating amplitude of the interference spectrum corresponds to the flatness of the epilayer, the smaller oscillating amplitude indicate that the surface of wafer A is likely to be rougher than that of wafer B. As verified by optical microscopic images in Fig. 5b, the surface morphology of wafer A is rich in identified steps, whereas that of wafer B is fairly smooth. The rougher surface in wafer A is further recognized by atomic force microscopy (AFM) consistent with the optical microscopic images, as shown in Fig. 5c, These results suggest that longer nitridation time at the initial growth stage is capable of adjusting the film flatness and introducing steps in the subsequent epilayer growth, which may effectively trigger the multidimensional SL formation.

**Figure 6:**
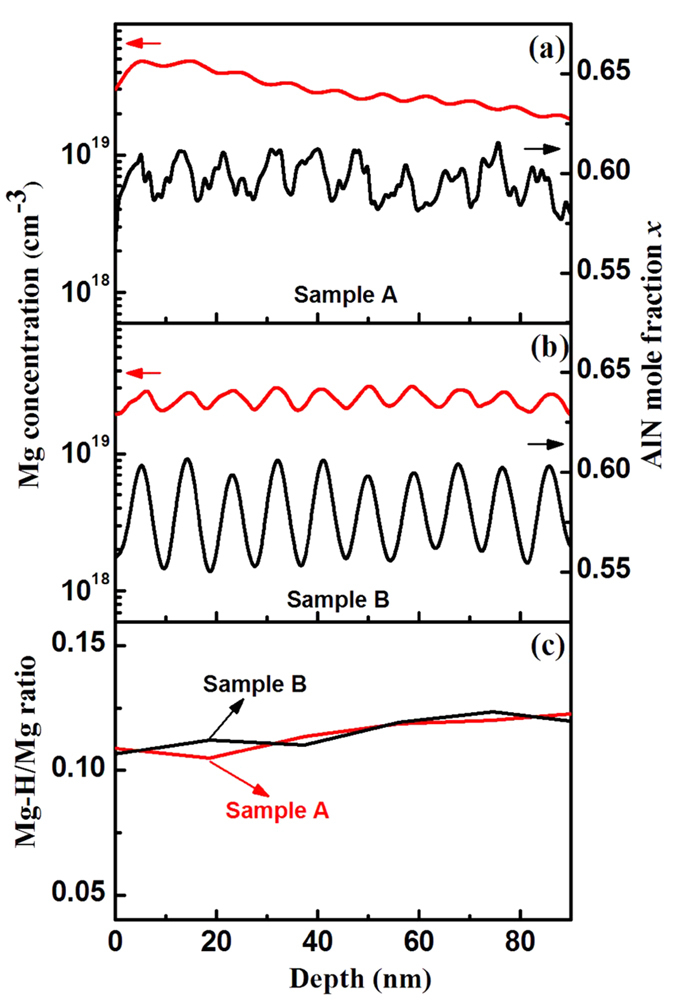
SIMS depth profiles of Mg, Al, and H atoms in sample A (**a**) and sample B (**b**). (**c**) Ratio between Mg–H concentration and Mg concentration.

To study the Mg concentration in SLs, the SIMS depth profiles of Mg and Al were carried out and compared between samples A and B [Fig. 6a,b]. Al concentration in both samples oscillated through the depth of 9 nm, which is in agreement with the designed period of SLs. The Mg concentration surges up and down at the interface between well and barrier, where ultimate V/III ratio was implemented to yield extremely N-rich conditions to enhance Mg incorporation referred to in our previous work^16^. Notably, the oscillation amplitudes of Al and Mg in sample A are smaller than those in sample B, which agrees with the multidimensional SL characteristics in sample A. With regard to the step morphology in Fig. 5b, the well and barrier are alternatively aligned on the in-plane grown layer, resulting in the weaker variation of the Mg and Al concentrations compared with those in conventional SLs. The effective incorporation of Mg is generally believed to be closely linked to the association between Mg and H concentrations^37^,^38^,^39^. To obtain a clear demonstration of the effective incorporation of Mg, the ratio between Mg–H and Mg concentrations is shown in Fig. 6c. Clearly, multidimensional SLs have the ratio similar to that of conventional SLs, indicative of the fact that Mg atoms are equally released from the bounded Mg–H complexes after annealing^40^ and activated as a *p*-type acceptor in AlGaN.

**Figure 7:**
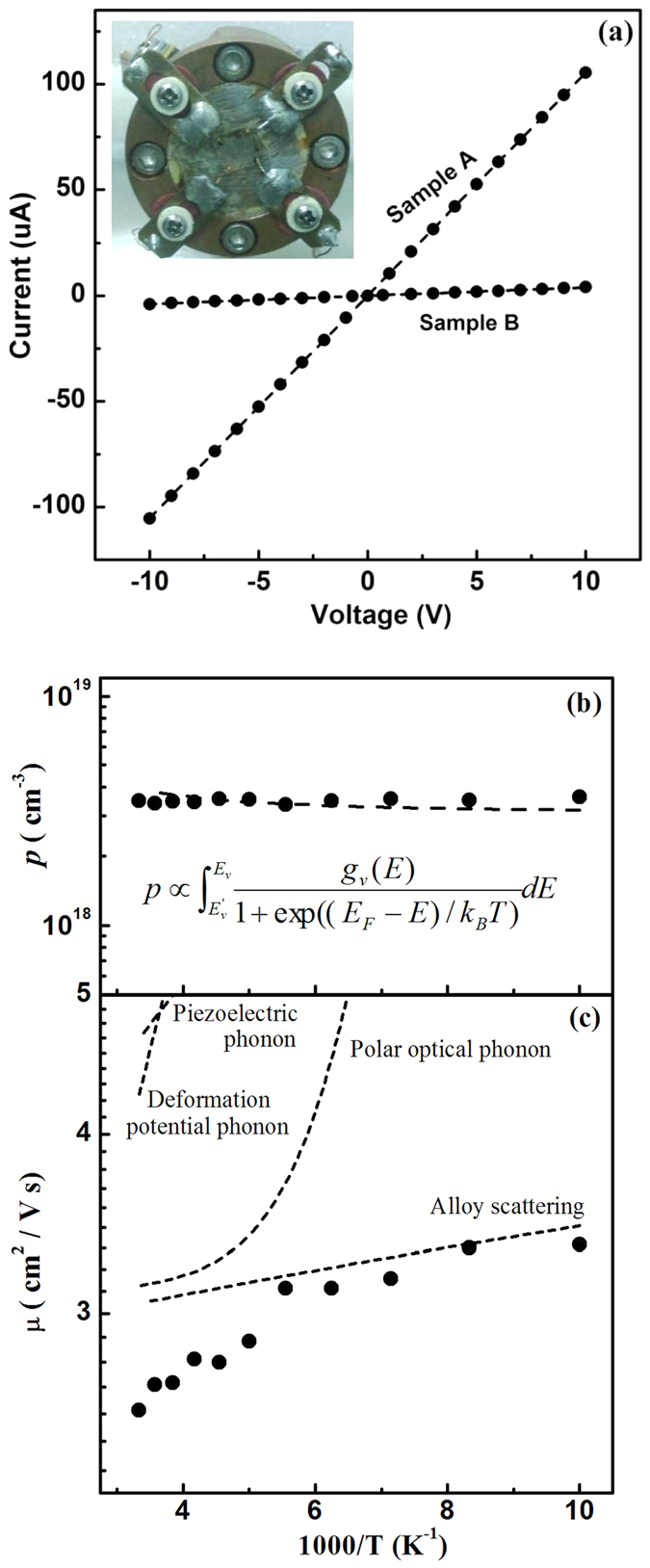
(**a**) *I-V* characteristics of samples together with the processed samples for Hall and *I-V* measurements shown in the inset. Hole concentration (**b**) and hole mobility (**c**) of sample A as a function of reciprocal temperature.

The fascinating aspect of conductivity is characterized by *I*-*V* measurement for samples A and B. The current scales linearly with the voltage that varies from −10 V to 10 V, indicating good Ohmic characteristics [Fig. 7a]. Sample A also exhibits good conductivity with the resistivity *ρ* as low as 0.7 Ω cm, which is characterized by van der Pauw resistivity measurement. Meanwhile, for sample B, the resistivity measured to be approximately 30 Ω cm, tens times higher than that of sample A. In general, the conductivity is described in terms of *σ* = 1/*ρ* = *nqμ*, where *n* is the carrier concentration, *q* is the carrier charge, and *μ* is the carrier mobility. Based on the Hall-effect measurements, the hole concentration of sample A is determined to be 3.5 × 10^18^ cm^−3^ at room temperature, whereas the Hall voltage of sample B is lower than the ultimate limit for hall measurement. Basically, in conventional SLs with in-plane structure, the hole in the film is likely to transport laterally. However, the multidimensional SLs with geometry structure in height enable vertical transports of holes before lateral transport among electrodes. Despite long-distance transport of holes in multidimensional SLs, the improved conductivity suggest the more efficient vertical hole transport. These results imply that the vertical barrier for holes plays a key role in conductivity in excellent agreement with the theoretical prediction.

To further understand the enhancement of conductivity, variable temperature Hall-effect measurements were performed on sample A in a temperature range from 100 K to 300 K, with 20 K increments. The symbols in Fig. 7b plot the temperature-dependent hole concentration of sample A. Note that the hole concentration is weakly dependent on temperature and can be maintained even at low temperature, which demonstrates the SL doping characteristics^27^. Hole is generally known to originate from thermal ionization, which is commonly determined by 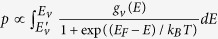. By using the *ab initio* calculated energies and the relevant DOS of the top valence bands, the experimental data in solid circles were fitted well in this formula [dashed lines in Fig. 7b], which lends support for the reduction of average activation energy in multidimensional SLs and their advantages for low-temperature applications. On the other hand, the hole mobility increases with decreasing temperature [symbols in Fig. 7c]. The observed trend deviates from impurity scattering and non-polar optical phonon scattering, indicating the insignificant effect of the referred scatterings^32^. Generally, the mechanism behind the trend possibly comes from a combination of polar optical phonon scattering, piezoelectric acoustic phonon scattering, acoustic phonon deformation potential scattering, and alloy scattering^35^,^36^. By fitting the temperature dependence of each scattering model [dashed lines in Fig. 7c] to experimental data, the hole mobility can not be explained solely by scattering model either. In this respect, the hole mobility of multidimensional SL behaves similar as bulk case. With advances in enhancing hole concentration and effectively reducing the scattering barrier in the vertical direction of multidimensional SLs, multidimensional SLs are capable of producing highly conductive *p*-type Al-rich AlGaN, which can pave a way to solve *p*-type doping difficulty.

## Conclusion

In summary, we put forward a novel multidimensional Mg-doped superlattice (SL) to enhance the vertical hole conductivity in conventional Mg-doped AlGaN SL because of the large potential barrier for holes. First-principles calculations show that the 3D Mg-doped SL decrease the hole potential barrier along the *c*-axis, because of the stronger *p*_*z*_ hybridization between Mg and N. Based on the theoretical results, highly conductive *p*-type 3D Al_0.63_Ga_0.37_N/Al_0.51_Ga_0.49_N SLs were grown with identified steps by increasing nitridation time at the initial stage of MOVPE. The hole concentration reaches a high value of 3.5 × 10^18^ cm^−3^ with a resistivity as low as 0.7 Ω cm at room temperature by Hall measurement, exhibiting tens times improvement in conductivity compared with that of conventional SLs. Furthermore, the resistivity is weakly dependent on temperature, and the high hole concentration can be maintained even at low temperature. The theoretical and experimental results clearly demonstrate the success of Mg-doped 3D SLs in producing highly conductive *p*-type Al-rich AlGaN, which can pave a way for achieving AlGaN-based deep UV devices with high performance.

## Methods

### Simulation

To explore the electrical properties of the Mg doped multidimensional SL, the 3D Mg-doped AlN/GaN and Al_0.75_Ga_0.25_N/Al_0.5_Ga_0.5_N SL as representative models were constructed on 256-atom (1 × 8 × 8) wurtzite supercells periodically repeated along [0001] to compare with conventional SL. The simulations were performed by means of the Vienna *ab initio* simulation package^41^. Pseudopotentials were specified using the projector augmented-wave^42^,^43^ method within the generalized gradient approximation^44^. The Ga *3d* electrons were treated as part of the valence basnd. The Brillouin zone was sampled with an 8 × 1 × 1 Monkhorst–Pack mesh^45^ and a cutoff energy of 520 eV was used for plane waves. All atomic degrees of freedom, including lattice parameters, were fully relaxed with electronic iteration convergence of 0.1 meV.

### Fabrication

The Mg-doped Al_*x*_Ga_1−*x*_N/Al_*y*_Ga_1-y_N SLs were grown on (0001) sapphire substrates via MOVPE (Thomas Swan 3 × 2 CCS). During the growth, trimethylgallium (TMGa), trimethylaluminum (TMAl), and ammonia (NH_3_) were used as precursors for Ga, Al, and N, respectively, with H_2_ as the carrier gas. Bis-cyclopentadienylmagnesium (Cp_2_Mg) was used as the *p*-type doping source. To modify the surface morphology, nitridation time was altered in the initial growth stage. For example, the nitridation lasted 400 and 200 s for wafers A and B respectively. The growth parameters remained the same for two samples by depositing a 20 nm-thick low-temperature AlN nucleation layer. After the growth of a 600 nm-thick AlN epilayer, 10 periods of AlN (3 nm)/Al_0.63_Ga_0.37_N (2 nm) SL interlayer was grown to release strain^46^ and filter threading dislocations which are expected to be lowered to 10^8^ cm^−2^ 47. The deposition was then followed by a 1.2 μm-thick Al_0.63_Ga_0.37_N layer. Subsequently, 12 periods of Mg-doped Al_0.63_Ga_0.37_N/Al_0.51_Ga_0.49_N SLs were grown at 1090 °C. The thicknesses of barriers and wells were set to 5 and 4 nm respectively, with the average Al composition of 0.58. To enhance efficient incorporation of Mg, the modified surface-engineering technique was applied during growth from well to barrier to realize an ultimate V/III ratio condition (extremely N-rich) by closing the metal flows (TMAl, TMGa, and Cp_2_Mg) and maintaining the NH_3_ flow for 2 s^16^. Finally, a 10 nm-thick Mg-doped GaN layer was grown to serve as a *p*-contact layer. After growth, wafers were annealed at 800 °C for 20 min in nitrogen atmosphere to activate Mg acceptors. Ni (20 nm)/Au (50 nm) *p*-type Ohmic contacts were initially deposited on cleaved 8 × 8 mm^2^ samples with the van der Pauw geometry which has the four symmetrically located points at a distance of 7 mm on the film surface using an e-beam evaporation system (VPT Citation3000), and were then annealed for 5 min in the air at 400 °C to convert their characteristics from rectifying to Ohmic.

### Measurements

The surface morphology of samples was characterized by optical microscopy (OLYMPUS BX51M) and atomic force microscope (AFM, Seiko SPA400). Secondary ion mass spectrometer (SIMS) measurements using the Cameca IMS 5FE7 system were conducted to determine Mg, Al, Ga, and H concentrations, with a depth resolution of approximately 1.5 nm and Cs^+^ ion beams as primary ion sources. The *I*-*V* electrical characteristics were measured using a Keithley 2450 Source Meter. To investigate the resistivity and hole concentration, variable temperature Hall-effect measurements were performed from 100 K to 300 K in 20 K increments, with a magnetic induction of 0.55 T.

## Additional Information

**How to cite this article**: Zheng, T. C. *et al.* Improved *p*-type conductivity in Al-rich AlGaN using multidimensional Mg-doped superlattices. *Sci. Rep.* **6**, 21897; doi: 10.1038/srep21897 (2016).

## Figures and Tables

**Figure 1:**
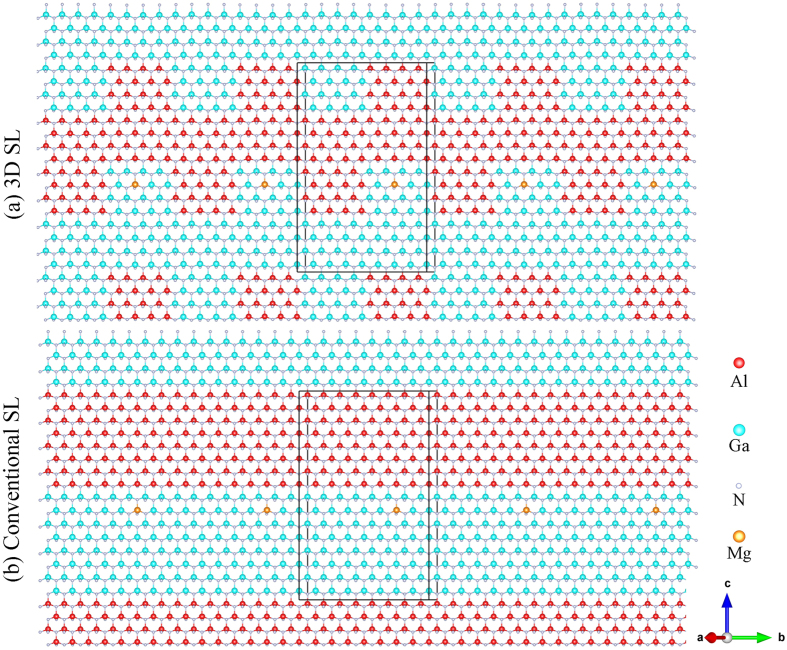
Crystal structures of 3D (**a**) and conventional AlN/GaN SL (**b**).
